# LINC00244 suppresses cell growth and metastasis in hepatocellular carcinoma by downregulating programmed cell death ligand 1

**DOI:** 10.1080/21655979.2022.2050073

**Published:** 2022-03-10

**Authors:** Zhijia Sun, Chunyuan Xue, Jiangbo Li, Hui Zhao, Yimeng Du, Nan Du

**Affiliations:** a Department of Oncology, Medical School of Chinese PLA, Beijing, Beijing, China; b Department of Genetic Engineering Lab, Beijing Institute of Biotechnology, Beijing, Beijing, China

**Keywords:** Hepatocellular carcinoma, long non-coding RNA, LINC00244, programmed cell death ligand 1, epithelial-mesenchymal transition

## Abstract

The role of programmed cell death ligand 1 (PD-L1) in suppressing antitumor immune responses has been widely reported, and recent studies showed that PD-L1 also plays an important role in epithelial-mesenchymal transition (EMT), determination of tumor cell phenotypes, metastasis, and drug resistance. Long non-coding RNAs (lncRNAs) are involved in a variety of epigenetic regulatory processes. The tumorigenesis and development of most cancers cannot be studied separately from their regulation by lncRNAs. To explore the epigenetic regulation of PD-L1, we identified an lncRNA, LINC00244, which reduced PD-L1 expression and predicted good clinical outcomes in hepatocellular carcinoma (HCC). LINC00244 inhibited the proliferation, invasion, and metastasis of HCC by downregulating PD-L1 expression. In addition, low LINC00244 expression activated epithelial-mesenchymal transition (EMT) pathways and facilitated the rapid growth and metastasis of HCC cells. Thus, LINC00244 is a potential therapeutic target for HCC.

## Graphical abstract

**Figure:**
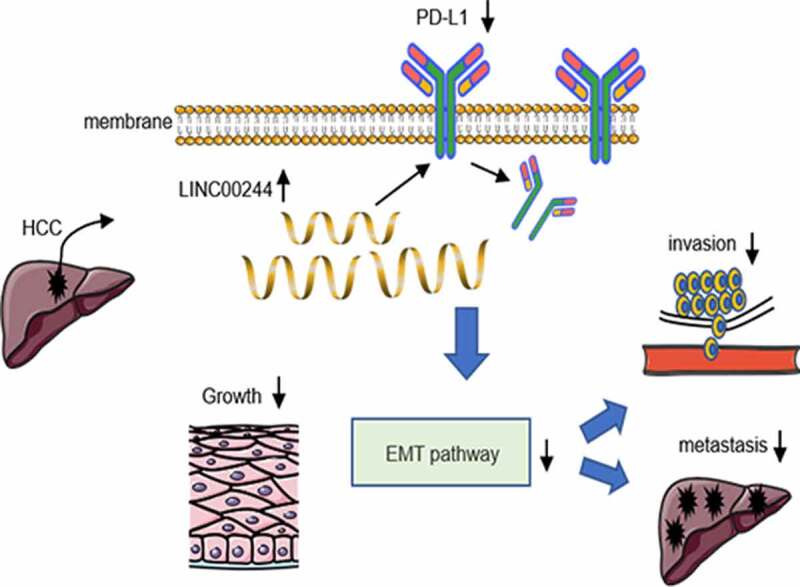

## 1. Introduction

Hepatocellular carcinoma (HCC) is the main histological type of liver cancer and has a very high recurrence and metastasis rate [1]. Owing to the clinically atypical symptoms of HCC, most patients are diagnosed when the disease is at a middle or advanced stages, and they therefore have few opportunities for surgery. Although targeted and interventional therapies provide survival benefits to patients with HCC, their prognosis is still not optimistic [2].

Programmed cell death ligand 1 (PD-L1), a member of the CD28 superfamily, plays an important role in the development and progression of HCC. Studies have reported that high PD-L1 expression is a predictor of disease recurrence in patients with HCC [3]. Analysis of HCC tumor tissue samples have shown that high PD-L1 expression is often closely associated with poor prognosis [4]. Consequently, high PD-L1 levels are not conducive to treatment and can cause remission of liver cancer. Previous studies focused on PD-L1ʹs immune checkpoint function, that is, PD-L1 transmits inhibitory signals by binding to programmed cell death 1 (PD-1) on the surface of T cells, leading to the immune escape of tumor cells [5]. As the intrinsic functions of PD-L1 are being discovered, scientists are turning increasing attention to how PD-L1 controls signaling within and between cancer cells [6]. In HCC, upregulation of PD-L1 promotes activation of the epithelial-mesenchymal transition (EMT)E pathway and leads to tumor growth and drug resistance [^7–9^].

Multiple long noncoding RNAs (lncRNAs) have been identified over the past few years. LncRNAs affect the regulation of chromatin modification, transcriptional activation and interference, and other cellular behaviors. To date, several lncRNAs have been reported as upregulating PD-L1 through various mechanisms, including regulating the expression of upstream and downstream gene regions, interfering with the shearing of target mRNA, and binding to specific proteins to regulate their activities or change their localization [10]. For example, Cao et al. reported that LINC00657 directly resulted in efficient PD-L1 expression in liver cancer cells [11]. The KCNQ1OT1 sponges miR-15a to promote the malignant progression of prostate cancer by upregulating PD-L1 [12]. However, few lncRNAs that downregulate PD-L1 expression have been identified. Negative PD-L1 regulation may inhibit HCC growth and metastasis, and thus present far-reaching significance for HCC treatment.

In this study, we screened different databases, including TCGA and Cbioporta, and identified one lncRNA, LINC00244, which could potentially negatively regulate PD-L1 expression and predict good clinical outcomes in HCC. We then investigated the effect of LINC00244 on the proliferation, invasion, and migration of hepatoma cells via regulation of PD-L1 expression. Here we attempt to provide theoretical and experimental evidence for potential therapeutic targets for HCC.

## 2. Materials and methods

### 2.1. Antibodies and reagents

The PD-L1 mouse monoclonal antibody (66248-1-Ig), E-cadherin polyclonal antibody (20874-1-AP), N-cadherin polyclonal antibody (22018-1-AP), and α-tubulin monoclonal antibody (66031-1-Ig) were obtained from Proteintech (Wuhan, Hubei, China). Vimentin rabbit monoclonal antibody (#5741), anti-mouse IgG antibody (ab190475), and anti-rabbit IgG antibody (ab205718) were purchased from Abcam (Shanghai, China).

### 2.2. RNA isolation and real-time PCR

After 48 h of transfection, total RNA was extracted from transfected cells using TRIzol RNA isolation reagent (Invitrogen, Waltham, MA, USA) according to the manufacturer’s instructions. A universal real-time PCR kit (Solarbio, Beijing, China) was used to reverse transcribe the isolated RNA into complementary DNA (cDNA). A fluorescence quantitative PCR instrument (CFX96 Touch; Bio-Rad) was used to measure gene expression. Real-time PCR was performed according to the manufacturer’s instructions. All procedures were performed on ice. Glyceraldehyde-3-Phosphate Dehydrogenase (GAPDH) (Bio-Rad, Hercules, CA, USA) was used as an internal reference gene, and each sample was processed in triplicate. Relative expression quantitative (RQ) values of different specimens were calculated using the 2− ΔΔCt method [13]. The primers used for real-time PCR are listed in Table 1.

**Table 1.** Primers used sequences for real-time PCR

**Gene**	**Species**	**Forward (5’→3’)**	**Reverse (5’→3’)**
PD-L1	Human	GCTGCACT AATTGTCTATTGGGA	AATTCGCTTGTAGTCGGCACC
LINC00244	Human	TTCCTCCATTTTAAAATTTTATTC	ATACAATGTAAGGATTTCCATAG
GADPH	Human	ACCCACTCCTCCACCTTTGAC	TGTTGCTGTAGCCA AATTCGTT
U6	Human	CTCGCTTCGGCAGCACA	AACGCTTCACGAATTTGCGT
18S	Human	GGCGCCCCCTCGATGCTCTTAG	GCTCGGGCCTGCTTTGAACACTCT

### 2.3. Cell culture, plasmids, and RNA oligonucleotides

Human liver cancer cell lines (HepG2 and Hep3B) were purchased from ATCC (Manassas, VA, USA). Both cell lines were cultured in Dulbecco’s Modified Eagle’s Medium (DMEM) supplemented with 10% fetal bovine serum (FBS). The cells were incubated at 37°C with 5% CO_2_. Eukaryotic expression vectors were generated by inserting PCR-amplified fragments into pcDNA3.1 vector (Transheep, Shanghai, China). LINC00244 promoter and LINC00244 mutant luciferase reporters were generated by amplifying the corresponding promoter fragment in the genomic DNA library and binding it to the pGL4.1 vector (Promega, Madison, WI, USA). The smart pool of LINC00244 and PD-L1 silencers was produced at RiboBio (Guangzhou, Guangdong, China). Lipofectamine RNAimax (Life Technologies) was used for transient siRNA transfection. The transfection reagent Vigorous (Vigorous Biotechnology, China) was used for the transient transfection of plasmids. Lentiviral vectors expressing PD-L1, LINC00244, or PD-L1 shRNA were constructed by cloning the PD-L1, LINC00244, or PD-L1 shRNA fragments into pCDH-EF1-MCS-T2A-Puro or pSIH-H1-Puro (System Biosciences), respectively. Stable cell lines overexpressing PD-L1, LINC00244, or PD-L1 shRNA were generated by infecting lentiviruses carrying PD-L1, LINC00244, or PD-L1 shRNA and selected using puromycin. The cDNA target sequences for the shRNAs and siRNAs are presented in Table 2.

**Table 2.** The cDNA target sequences of shRNAs or siRNAs

**Gene**	**Target sequence (5’→3’)**
LINC00244 (siRNA1)	CCAAGGTAAATGGAGCTAA
LINC00244 (siRNA2)	GAATCCTAGGCATTCGTGT
LINC00244 (siRNA3)	CACATAGTAGCCACACGAA
PD-L1 (siRNA)	CGAATTACTGTGAAAGTCAAT
PD-L1 (shRNA)	GATCCCGAATTACTGTGAAAGTCAATCTTCCTGTCAGAATTGACTTTCACAGTAATTCGTTTTTG

### 2.4. Western blotting

After digesting the cells with trypsin, they were collected and washed twice with 1× PBS and lysed on ice for 30 min using radioimmunoprecipitation assay lysis buffer. Next, 1× loading buffer was added to the lysed product and the mixture was placed in a 100°C water bath for 15 min. Subsequently, the supernatant containing target proteins was run on an SDS-PAGE gel, after which the separated proteins were transferred onto nitrocellulose filter membranes. Tris-buffered saline containing 1% Tween 20 (TBST) supplemented with 5% skimmed milk powder was used to block nonspecific protein binding. After incubation with the primary antibodies on a rotary shaker for 1.5 h, the membranes were washed three times with 1× TBST for 5 min each. HRP-conjugated secondary antibodies were then used to bind the primary antibodies for 1 h. Finally, ECL Luminous Liquid (Millipore) was used to develop the target protein band and the images analyzed using Image Lab^TM^ software (Bio-Rad).

### 2.5. Detection of cell surface PD-L1

PD-L1 on the cell surface was detected as previously described [14]. Briefly, cells transfected with LINC00244 or empty vector were seeded onto small dishes and allowed to grow to 80% confluence. All cells were trypsinized and washed twice with 1× PBS. Next, the cells were suspended in cell staining buffer containing APC anti-human PD-L1 mAb antibody (1:100) and incubated in the dark at 25°C for 30 min. The cells were then washed three times with ice-cold cell staining buffer and re-suspended in a 300 µL staining buffer for detection.

### 2.6. Cell proliferation assay

Cells transfected with the designated plasmids were inoculated into a 96-well flat-bottom plate at a density of approximately 3,000 cells per well. The CCK-8 kit (Dojindo, Kyushu, Japan) was used to evaluate the proliferation of the transfected cells according to the manufacturer’s instructions [15].

### 2.7. Colony formation experiment

Cells transfected with the designated plasmids were inoculated into 6-well cell culture dishes and the number of cells per well maintained at approximately 1000. The cells were stained with crystal violet after culturing for approximately 10–14 days. Colonies larger than 1.5 mm in diameter were included in the clone formation count.

### 2.8. Cell invasion and migration experiments

Cells were cultured in Transwell chambers with DMEM. Complete DMEM containing FBS was added to the lower chamber. Cell invasion experiments were conducted according to the manufacturer’s instructions (BD Biosciences, NY, USA) [16].

To measure cell migration, the transfected cells were cultured in 60-mm diameter dishes. Once the cells reached 90% confluency, a 200 μL sterile pipette tip was used to scrape off the cells in a line in the center of the dish and an inverted microscope (OLYMPUS. Japan) was used to capture images. Cells were photographed again after 24 h of incubation to compare cell migration.

### 2.9. Separation of nuclear and cytoplasmic fractions

HepG2 and Hep3B cells were collected once they had reached 90% confluence and lysed in a lysis buffer (5 mM KCl, 1 mM MgCl_2_, 25 mM Tris-HCl, and 1% NP-40; pH 7.4) on ice for 10 min. The lysate was centrifuged at 1500 g and 4°C for 4 min. The resulting supernatant contained cytoplasmic elements. The pellet was resuspended in nuclear recovery buffer (20 mM N-2-hydroxyethylpiperazine-N-ethane-sulphonicacid pH 7.9, 1 mM EDTA, 1 mM EGTA, 400 mM NaCl, 1 mM PMSF, and 1 mM DTT) for 30 min and subjected to high-speed centrifugation at 13000 g and 4°C for 10 min. The resulting supernatant contained the cell nuclei. RNA was extracted from the cytoplasm and nuclei. 18S rRNA was used as a cytoplasmic RNA marker, while U6 rRNA was used as a nuclear RNA marker.

### 2.10. Tissue sample collection and immunohistochemistry (IHC)

A total of 25 HCC samples were collected from participants recruited at the Beijing People’s Liberation Army General Hospital and processed by IHC and fluorescence in situ hybridization (FISH). The study was approved by the Research Ethics Committee of the Forth Medical Center of Chinese PLA General Hospital (No. 2020KY007-KS002). The methodology conformed to the standards set out in the Declaration of Helsinki. All participants were informed of the study design and signed an informed consent form. All IHC results were analyzed independently by two pathologists who had no information on the HCC patients. A histochemistry score was generated by adding 3× the percentage of strongly stained cells, 2× the percentage of moderately stained cells, and 1× the percentage of weakly stained cells [17].

Tissue specimen were dewaxed, rehydrated, and subjected to antigen retrieval for 30 min using citrate in a pressure cooker containing boiled water. After treatment with 3% H_2_O_2_ for 15 min, the samples were incubated overnight at 4°C with anti-PD-L1 (1:500, Proteintech, 66248-1-Ig). After incubation with a universal secondary antibody for 1 h at 37°C and washing three times with 1× PBS for 5 min, 3,3 N-Diaminobenzidine Tetrahydrochloride was used to develop visualization signal.

### 2.11. Fluorescence in situ hybridization (FISH)

The cells were attached firmly on confocal dishes with paraformaldehyde (4%) and permeabilized with Triton 100 (0.5%) at 4°C for 30 min. Next, 200 μL of Prewarmed prehybridization liquid was added into each dish and incubated for 1 h at 37°C. Cells in each dish were incubated in the dark with 100 μL hybridization solution containing 2 μL hybridization probes at 37°C for 4 h. After incubation, the dishes were successively washed with 4×, 2×, and 1× sodium citrate buffer heated to 42°C, followed by a single wash with 1× PBS for 5 min at 25°C. DAPI was used to stain the cells. Finally, the cells were visualized under a fluorescence microscope (TH4-200; Olympus).

For HCC tissues, lncRNA FISH was performed on paraffin tissue sections using a probe specific for human LINC00224, according to the manufacturer’s instructions (RiboBio, China). An immunofluorescence microscope (TH4-200, Olympus, Japan) was used to capture images.

### 2.12. Generation of a mouse model and immunoassay

The experimental animal research protocol was approved and supervised by the Ethics Committee of the Beijing Institute of Biotechnology. A mouse model of liver cancer was established using nude mice. Briefly, 1 × 10^7^ HepG2 cells loaded with the designated constructs were subcutaneously injected into the left back of nude mice (age: 4–6 weeks, body weight: 18–22 g). The control group was inoculated with HepG2 cells transfected with EV, while the two experimental groups were inoculated with HepG2 cells transfected with either LINC00244 or LINC00244 plus PD-L1. There were 7 mice in each group. Tumor size was measured every four days using calipers. Tumor volume was computed as: volume = (length × width^2^)/2 [18]. The mice were sacrificed 40 days after injection. Isolated tumors were stored at −80 °C and processed for IHC and LINC probe hybridization.

### 2.13. Statistical analysis

SPSS (version 23.0) and GraphPad Prism (version 8.0) software were used to analyze the experimental data. Quantitative data are presented as mean ± standard deviation. Data were compared using Student’s t-test. Spearman correlation coefficient was used for multivariate analysis. All in vitro tests were conducted in triplicate. Differences were considered statistically significant at *p*< 0.05.

## 3. Results

We screened three databases for lncRNAs that are negatively correlated with PD-L1 and associated with a good HCC prognosis. We identified LINC00244 as the candidate lncRNA based on result of western blot and RT-PCR tests. We then validated the effect of LINC00244 on hepatoma cells using several cell function-related experiments and observed similar effects in vivo. Finally, using gene enrichment analysis, we found that LINC00244 is associated with the PD-L1-mediated EMT pathway. These results were verified using western blotting assays. Our results showed that LINC00244 is a promising target for the treatment of HCC.

### 3.1. LINC00244 negatively regulates PD-L1 and is linked with good HCC prognosis

**Figure 1.**
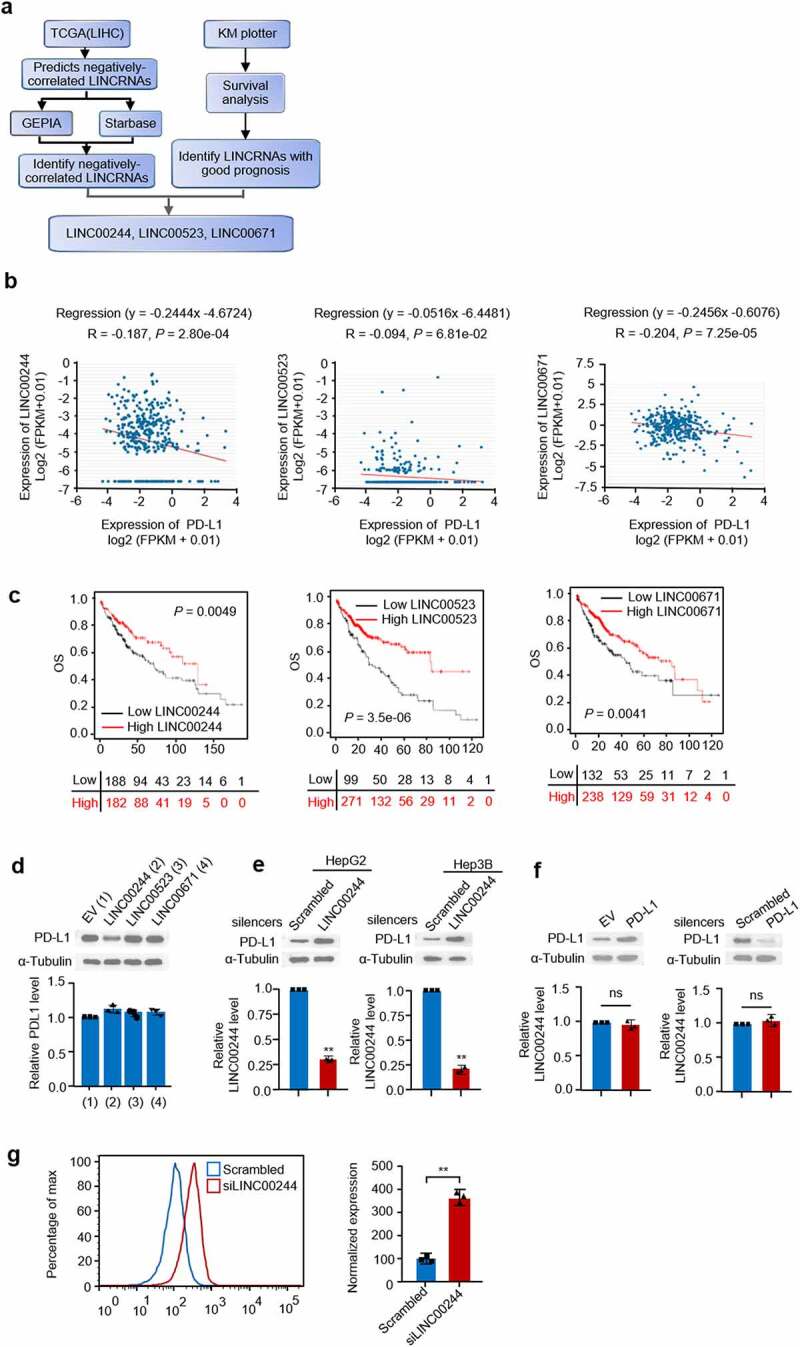
LINC00244 can negatively regulate programmed cell death ligand 1 (PD-L1) and is associated with good hepatocellular carcinoma (HCC) prognosis. (a) Diagram showing the screening process used to identify lncRNAs that negatively regulate PD-L1 and predict good prognosis. (b) Pearson correlation analysis shows the degree of correlation between PD-L1 and lncRNAs (https://starbase.sysu.edu.cn). (c) The overall survival rate of patients with HCC with low or high lncRNA expression were analyzed using Kaplan-Meier (http://kmplot.com/analysis). (d) RNA and protein levels of PD-L1 were detected using RT-PCR and western blot, respectively, following transfection with empty plasmid, LINC00244, LINC00523, or LINC00671. (e) Expression levels of LINC00244 and PD-L1 in HepG2 and Hep3B cells following transfection of scrambled or LINC00244 smart pool of silencers. (f) Expression levels of LINC00244 and PD-L1 in HepG2 cells following transfection of PD-L1 or PD-L1 smart pool of silencers.(g) PD-L1 levels on the surface of HepG2 cells were detected by flow cytometry following transfection of scrambled or LINC00244 siRNA.

To identify lncRNAs that negatively regulate PD-L1 and predict good HCC prognosis, we summarized information from databases containing data on liver cancer based on the screening pattern shown in Figure 1(a). First, we used The Cancer Genome Atlas (TCGA; LIHC) to screen for lncRNAs that were negatively correlated with PD-L1. Data related to these candidate lncRNAs were compared with data from GEPIA and Starbase (Figure 1(b)). The lncRNAs that exhibited good clinical outcomes were identified using KM plotter (Figure 1(c)). Three lncRNAs (LINC00244, LINC00523, and LINC00671) were shown to both have negative correlation with PD-L1 and predicted good prognosis. The liver cancer cell line HepG2 was transfected with the three lncRNAs to investigate their potential to downregulate PD-L1 expression. Only LINC00244 resulted in a reduction in PD-L1 protein levels, while PD-L1 mRNA levels were not affected by any of the three candidate lncRNAs (Figure 1(d)). This suggests that LINC00244 downregulates PD-L1 expression in HepG2 cells. To further confirm the role of LINC00244 in HCC, we knocked down LINC00244 in two HCC cell lines (HepG2 and Hep3B) with siLINC00244, and western blot showed that PD-L1 expression significantly increased in both cell lines (Figure 1(e)). Similarly, previous studies showed changes in PD-L1 level using western blot to demonstrate PD-L1 regulation factors in HepG2 and Hep3B cells [7,19]. To confirm the effect of LINC00244, flow cytometry was performed. The result showed that PD-L1 levels on the surface of HepG2 cells increased when LINC00244 was knocked down (Figure 1(g)). To investigate the reverse effect of PD-L1 on LINC00244, PD-L1 was either overexpressed or knocked down in HepG2 cells. However, PD-L1 did not change intracellular LINC00244 levels (Figure 1(f)). This suggests that LINC00244 is the upstream regulatory factor for PD-L1 and that PD-L1 does not affect LINC00244.

### 3.2. LINC00244 inhibits proliferation, invasion, and migration by downregulating PD-L1 expression in HCC cells

**Figure 2.**
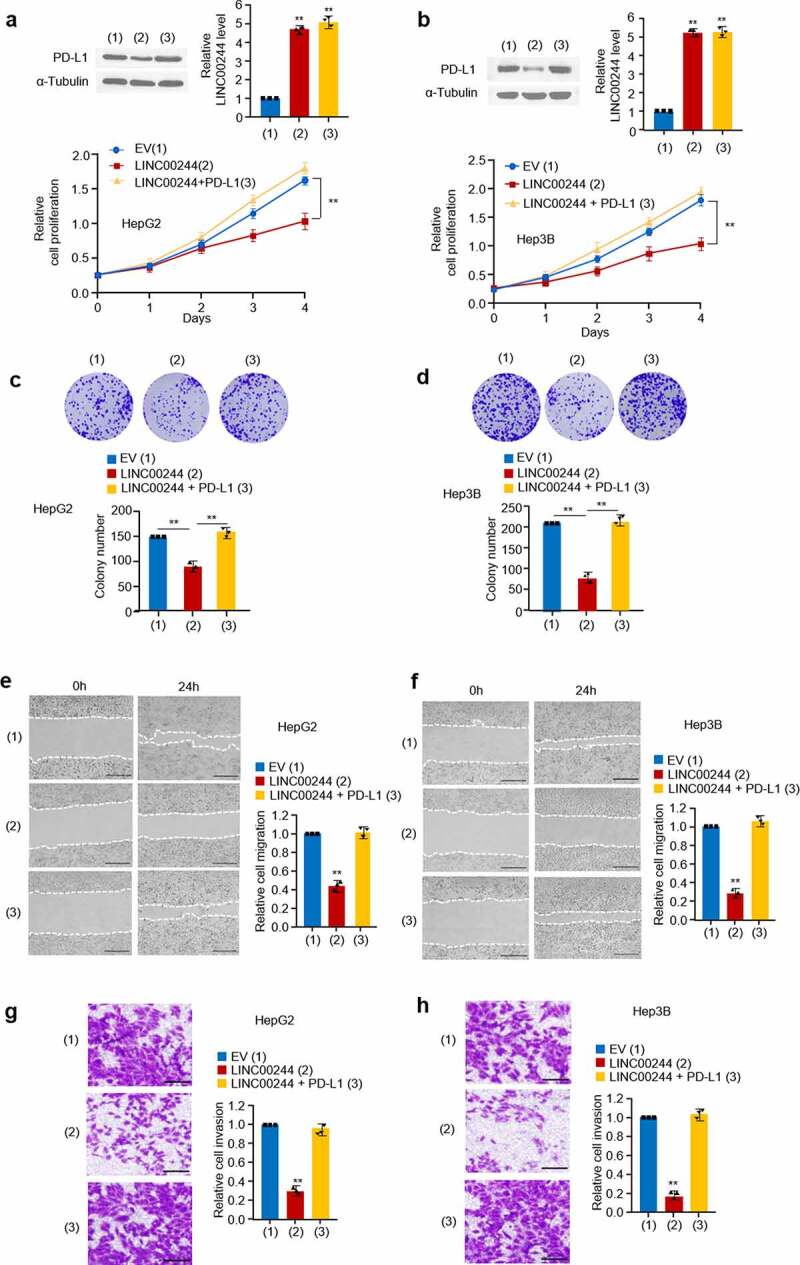
LINC00244 suppresses proliferation, invasion, and migration by upregulating programmed cell death ligand 1 (PD-L1) expression in hepatocellular carcinoma (HCC) cells. (a, b) HepG2 and Hep3B cells were transfected with either LINC00244 or LINC00244 plus PD-L1 expression vectors. Cell proliferation was detected using CCK-8 assay. The representative immunoblot shows PD-L1 expression. The histograms show LINC00244 expression as determined by RT-PCR. (c, d) Colony-formation assay of HepG2 cells transfected as in (a) and Hep3B cells transfected as in (b). The representative images show the colonies in the plate (above). The histograms show colony numbers. (e, f) Wound healing of HepG2 cells transfected as in (a) and Hep3B cells transfected as in (b). Scale bar, 100 μm. Right histograms show relative cell migration. (g, h) Transwell assay of HepG2 transfected as in (a) and Hep3B cells transfected as in (b). Scale bar, 500 μm. Right histograms show relative cell invasion. All displayed values represent mean ± SD. Measurements were taken in triplicate and gave similar results *p < 0.05, **p < 0.01 versus corresponding controls).

To study the biological role of LINC00244 in HCC, we overexpressed LINC00244 in HepG2 and Hep3B cells and measured cell growth, invasion, and migration. Cell proliferation and colony formation assays revealed that, compared with untreated cells, LINC00244 overexpression significantly affected the proliferation of HepG2 and Hep3B cells, while recovery of PD-L1 reversed these effects (Figure 2(a,d)). In addition, cell invasion and migration assays revealed that LINC00244 overexpression caused a significant decrease in the migration and invasion abilities of HepG2 and Hep3B cells. Similarly, we found this could be reversed by re-expressing PD-L1 (Figure 2(e–h)).

Next, we knocked down LINC00244 in PD-L1-silenced HepG2 cells and found that PD-L1 levels, cell proliferation, and wound healing did not change significantly (Figure S1 (a, b)). This result suggests that LINC00244 has no effect on cell growth in the absence of PD-L1. In summary, the above experiments demonstrated that LINC00244 alters the proliferation, invasion, and migration of HCC cells by regulating PD-L1 expression.

### 3.3. LINC00244 inhibits the growth of HCC cells in vivo by regulating PD-L1 expression

**Figure 3.**
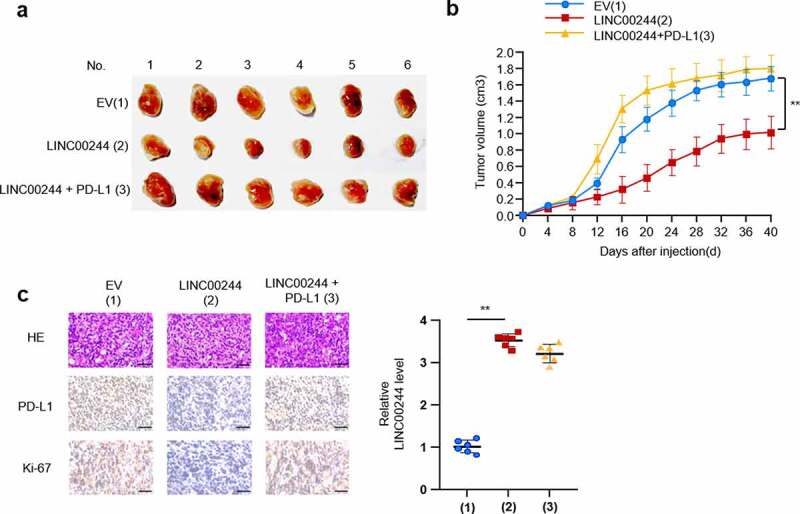
LINC00244 inhibits the growth of liver cancer cells in vivo by downregulating programmed cell death ligand 1 (PD-L1). (a) After stably infecting HepG2 cells with lentivirus carrying the specified construct, 106 cells were subcutaneously injected into nude mice (n = 6 per group). The picture shows dissected tumor tissue. (b) Tumor volume was measured every four days and the growth curve was generated using GraphPad. (c) Representative immunohistochemistry (IHC) staining of HE, PD-L1 and Ki67 of tumors resected from (a). Scale bar, 50 µm.

To confirm the influence of the LINC00244/PD-L1 axis on liver cancer *in vivo*, we explored the influence of this axis on the growth of liver cancer cells by inoculating HepG2 cells containing plasmid DNA constructs into the left back of nude mice. As predicted, LINC00244 inhibited the growth of HCC cells. In contrast, when PD-L1 was overexpressed, the effect of LINC00244 on tumor growth was significantly reduced (Figure 3(a,b)). The expression of Ki67 is closely associated with the proliferation and growth of tumor cells and is widely used as a proliferation marker in routine pathological examinations [20]. Compared with the IHC results from the control group, PD-L1 expression was lower in the group overexpressing LINC00244, and the proliferative ability of tumor cells was weakened. When PD-L1 expression was recovered, cell proliferation was also restored. (Figure 3(c)).

### 3.4. Correlation between LINC00244 and PD-L1 expression in human HCC

**Figure 4.**
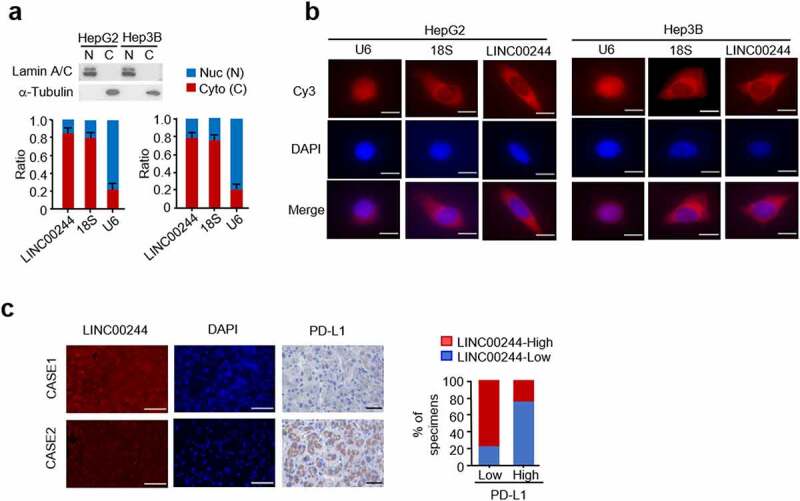
The localization of LINC00244 in hepatocellular carcinoma (HCC) cells and correlation between LINC00244 and programmed cell death ligand 1 (PD-L1) expression in patients with HCC. (a) LINC00244 expression in HepG2 and Hep3B cells was detected using RT-PCR. The separated nucleus and cytoplasm fractions were assessed using Western blot assays. The nuclear marker is Lamin A/C and the cytoplasmic marker is tubulin. (b) Subcellular localization of LINC00244 (red) in HepG2 and Hep3B cells was observed using fluorescence in situ hybridization (FISH). The nuclei were stained with 4’,6-diamididine-2’-phenylindole dihydrochloride (DAPI) (blue). Scale bar, 10 μm. (c) Representative IHC staining for PD-L1 and FISH staining for LINC00244 in patients with HCC. Scale bar, 50 μm. The histograms show the percentage of samples with low or high PD-L1 expressions in the low or high LINC00244 expression groups. CASE 1 and CASE 2 are two representative samples classified based on high or low LINC00671 expression.

We studied the intracellular localization of LINC00244, a newly identified lncRNA associated with liver cancer, in HCC cells. Real-time PCR was conducted following the extraction of nuclear and cytoplasmic components. The results revealed that LINC00244 is primarily located in the cytoplasm of both HepG2 and Hep3B cells (Figure 4(a)). These results were verified using RNA FISH experiments (Figure 4(b)). We also evaluated the expression of PD-L1 and LINC00244 in 16 clinical HCC samples. These results were consistent with the cellular and animal experimental results; LINC00244 expression was negatively correlated with PD-L1 expression (Figure 4(c)).

### 3.5. Low LINC00244 expression activates the EMT pathway in HCC cells and enhances infiltration and metastasis in HCC

**Figure 5.**
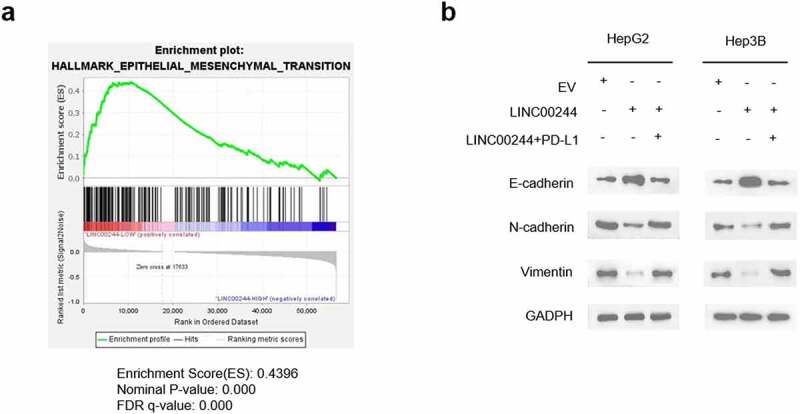
Low LINC00244 expression can promote the activation of the epithelial-mesenchymal transition (EMT) pathway in hepatocellular carcinoma (HCC). (a) Gene set enrichment analysis showed that LINC00244 levels were negatively correlated with the EMT pathway in TCGA (LIHC) dataset. (b) The left image shows the expression levels of E-cadherin, N-cadherin, and vimentin in HepG2 cells transfected with EV, LINC00244 or LINC00244 and PD-L1 expression vectors. The graph on the right shows the expression levels of the proteins in Hep3B cells transfected with the above vectors.

To further understand the mechanism through which LINC00244 regulates the invasion and metastasis of HCC cells, we analyzed gene set enrichment using HCC data from TCGA [21]. The results showed a negative correlation between the EMT pathway and LINC00244 expression (Figure 5(a)). Furthermore, we verified the effect of LINC00244 on the levels of the key EMT proteins E-cadherin, N-cadherin, and vimentin. As expected, when LINC00244 was overexpressed, E-cadherin expression significantly increased, whereas N-cadherin and vimentin expression were notably reduced. These protein changes indicate a suppression of the EMT pathway, as previously reported [22]. However, the expression levels of E-cadherin, N-cadherin, and vimentin proteins were comparable to those in the control cells following supplementation with intracellular PD-L1 (Figure 5(b)). In conclusion, LINC00244 significantly suppressed the EMT pathway by downregulating PD-L1.

## 4. Discussion

PD-L1 was first identified in 1999 as an immune checkpoint molecule that causes immune escape of tumor cells by binding to PD-1. Since then, other studies have identified several novel biological functions of PD-L1, although most of them are not associated with PD-1 [6]. Recent studies have found that PD-L1 can affect growth and survival of cancer cells, DNA damage response, and gene regulation. Qiu et al. demonstrated that intracellular PD-L1 confers glioblastoma multiforme cell malignancy and aggressiveness via binding Ras and activating the down-stream Erk-EMT signaling [23]. Tu et al. reported that PD-L1 can be targeted to sensitize to radiation or chemotherapy by regulating the stability of DNA damage related genes [24]. EMT is one of the main pathways through which PD-L1 regulates cancer cells. Chen et al. reported that the intracellular domain of PD-L1 can promote tumor metastasis by promoting the EMT pathway in triple-negative breast cancer cells [25].

PD-L1 regulation is a causal factor in the outcomes of various cancers. Heterogeneous Nuclear Ribonucleoprotein L (HNRNPL) can accelerate the growth and development of colorectal cancer tumors by upregulating PD-L1 expression [26]. Jiao et al. reported that IFI16 positively regulates PD-L1 expression in the STING-TBK1-NF-kB pathway, promoting the progression of cervical cancer [27]. In HCC, CircCORO1C enhances PD-L1 expression through the NF-κB pathway and promotes tumor proliferation and metastasis [19]. To inhibit tumor growth and metastasis, researchers are exploring compounds and natural molecules that can significantly downregulate PD-L1 expression. Berberine, a small-molecule compound, reduces PD-L1 expression in cancer cells and promotes anti-tumor immunity by inhibiting the deubiquitylation activity of CSN5 [28]. Shirin et al. reported that microRNA-383-5p limited breast cancer cell proliferation and migration and induced cell apoptosis by decreasing PD-L1 protein levels [29]. Xiao et al. proved that the naturally-modified molecule EZH2 can lower PD-L1 expression by directly upregulating the levels of the promoter H3K27me3 in HCC, providing a promising strategy for HCC immunotherapy [30]. Therefore, developing factors that can downregulate PD-L1 is of great significance for the study of cancer therapy.

LncRNAs are some of the most important molecules that regulate the expression level of proteins and subsequently influence biological cellular behavior. Many studies have shown that several lncRNAs can promote tumor growth and metastasis by upregulating PD-L1 expression. The lncRNA PSMB8-AS1 upregulates the expression of STAT1 by sponging miR-382-3p to increase the level of PD-L1, resulting in the promotion of proliferation, invasion, and migration in pancreatic cancer [31]. The lncRNA EMX2OS can bind directly to miR654 and inhibit its expression, leading to the upregulation of PD-L1 and the promotion of proliferation, invasion, and spheroid formation in ovarian cancer cells [32]. The lncRNA PSMA3 can upregulate PD-L1 and thus promote the progression and metastasis of bladder cancer [33]. All the lncRNAs mentioned positively regulated PD-L1 expression. However, studies on lncRNAs that negatively regulate PD-L1 expression are lacking. In this study, we discovered a novel lncRNA, LINC00244, that inhibits the proliferation, invasion, and metastasis of HCC by negatively regulating PD-L1 expression. This finding provides a new therapeutic target for the development of a promising clinical strategy to treat HCC.

Additionally, we demonstrated that LINC00244 can significantly suppress the EMT pathway by regulating PD-L1 expression. This is consistent with previous studies which showed that PD-L1 can activate the EMT pathway and accelerate tumor progression [34,35]. EMT is the process by which epithelial cells acquire the ability to infiltrate and migrate under the action of specific factors and transform into cells with an interstitial phenotype. It is one of the conditions necessary for tumor metastasis [36]. The expression of EMT molecules that improve cell adhesion and polarity (such as E-cadherin) are reduced, whereas the expression of molecules that enable cells to acquire infiltration and metastatic characteristics (such as N-cadherin and vimentin) are elevated [37]. To date, several lncRNAs have been closely associated with the EMT pathway. Zhang et al. reported that several lncRNAs can regulate the occurrence and development of kidney renal clear cell carcinoma by targeting EMT transcription factors [38]. Lei et al. reported that the upregulation of TUG1 can promote the EMT pathway in thyroid cancer [39]. Zhang et al. demonstrated that SNHG3 overexpression can induce EMT in HCC cells by activating the miR-128/CD151 cascade, leading to sorafenib resistance [40]. In this study, we identified LINC00244, a new lncRNA. Gene set enrichment and western blot analysis revealed that low LINC00244 expression promoted EMT. In addition, by recovering PD-L1 in LINC00244-overexpressing cell lines, EMT pathway activity was restored. Consequently, it is reasonable to hypothesize that LINC00244 inhibits the activity of the EMT pathway by reducing PD-L1 expression, thus inhibiting the proliferation, invasion, and migration of HCC cells.

Although PD-L1 inhibitors have demonstrated some efficacy in the treatment of HCC, their therapeutic effects are still limited due to drug resistance. Drug resistance can be caused by several factors, with increased PD-L1 expression being one of the most important [41]. Multiple mechanisms may lead to overexpression of PD-L1 in tumor cells, resulting in tumor growth and metastasis [42,43]. LINC00244 inhibits the growth of tumor cells by reducing PD-L1 expression. In summary, LINC00244 may be a promising target for the treatment of HCC.

## 5. Conclusion

We discovered a novel lncRNA molecule, LINC00244, that is associated with a good prognosis in HCC, and verified that it can inhibit the proliferation, invasion, and migration of tumor cells by reducing PD-L1 protein levels. LINC00244 overexpression significantly inhibited the EMT pathway, although this phenomenon could be reversed by overexpressing PD-L1. The current study did not identify the mechanism through which LINC00244 downregulates PD-L1 nor its complex molecular interactions with the EMT pathway. These will need to be investigated in future studies to provide additional evidence in support of the therapeutic effect of LINC00244.

## Supplementary Material

Supplemental Material
