# Supplementary figures and images for: LINC00244 suppresses cell growth and metastasis in hepatocellular carcinoma by downregulating programmed cell death ligand 1
Source: Bioengineered. 2022 Mar 10;13(3):7635–47. doi: 10.1080/21655979.2022.2050073 (PMC8974003; doi:10.1080/21655979.2022.2050073)

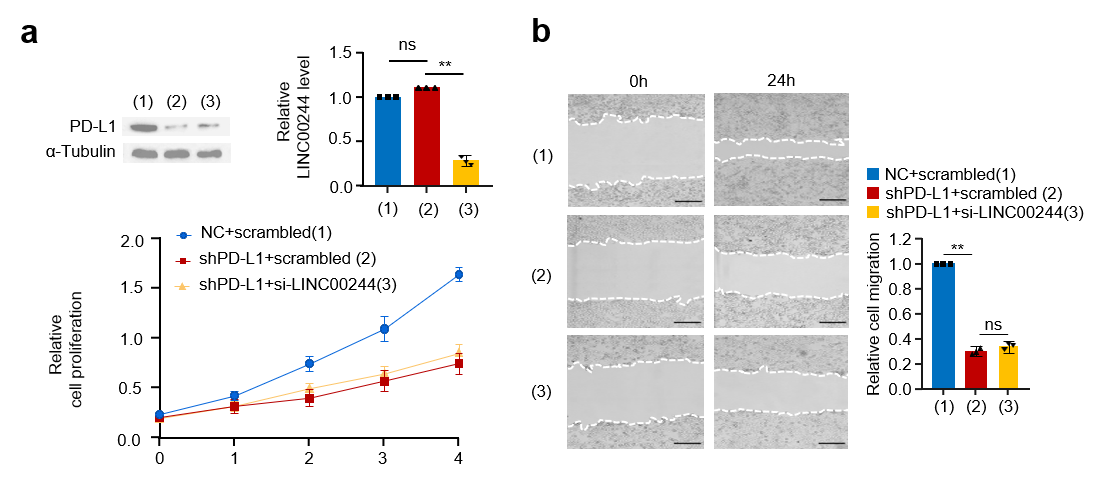

Supplement: Supplemental Material [file KBIE_A_2050073_SM5784.tif]
